# *In ovo* Feeding as a Tool for Improving Performance and Gut Health of Poultry: A Review

**DOI:** 10.3389/fvets.2021.754246

**Published:** 2021-11-11

**Authors:** Razib Das, Pravin Mishra, Rajesh Jha

**Affiliations:** Department of Human Nutrition, Food and Animal Sciences, College of Tropical Agriculture and Human Resources, University of Hawaii at Manoa, Honolulu, HI, United States

**Keywords:** chicken, embryo, gut health, *in ovo* technology, growth performance

## Abstract

Early growth and development of the gastrointestinal tract are of critical importance to enhance nutrients' utilization and optimize the growth of poultry. In the current production system, chicks do not have access to feed for about 48–72 h during transportation between hatchery and production farms. This lag time affects early nutrient intake, natural exposure to the microbiome, and the initiation of beneficial stimulation of the immune system of chicks. *In ovo* feeding can provide early nutrients and additives to embryos, stimulate gut microflora, and mitigate the adverse effects of starvation during pre-and post-hatch periods. Depending on the interests, the compounds are delivered to the embryo either around day 12 or 17 to 18 of incubation and via air sac or amnion. *In ovo* applications of bioactive compounds like vaccines, nutrients, antibiotics, prebiotics, probiotics, synbiotics, creatine, follistatin, L-carnitine, CpG oligodeoxynucleotide, growth hormone, polyclonal antimyostatin antibody, peptide YY, and insulin-like growth factor-1 have been studied. These compounds affect hatchability, body weight at hatch, physiological functions, immune responses, gut morphology, gut microbiome, production performance, and overall health of birds. However, the route, dose, method, and time of *in ovo* injection and host factors can cause variation, and thereby inconsistencies in results. Studies using this method have manifested the benefits of injection of different single bioactive compounds. But for excelling in poultry production, researchers should precisely know the proper route and time of injection, optimum dose, and effective combination of different compounds. This review paper will provide an insight into current practices and available findings related to *in ovo* feeding on performance and health parameters of poultry, along with challenges and future perspectives of this technique.

## Introduction

In the modern poultry production system, the first reported chick embryos injection of thiourea was done by Grossowicz in 1946 to observe the effect in hatchling and post-hatch life (1), followed by thyroxin by Balaban and Hill (2). Later, *in ovo* technique (IOT) was first opted for vaccination against Marek's disease by Sharma and Burmester (3). Subsequently, the success of *in ovo* vaccination (IOV) has set forth a paradigm shift in the poultry industry by adapting IOT to harness more benefits by changing the embryonic milieu and improving the nutritional conditions of neonatal chicks. Besides IOV, this technology is being utilized with ramifications to deliver growth promoting compounds and nutrients at the embryonic stage (*in ovo* feeding, IOF) (4), to improve the performance and gut health of poultry (5), to determine the sex of the embryo (*in ovo* sexing) (6), and to initiate epigenetic changes that improve the health and production status of the poultry at post-hatch period (7). The term IOF, *in ovo* stimulation, *in ovo* injection (IOI), *in ovo* delivery (IOD), and *in ovo* supplementation (IOS) are often interchangeably used as they are not well-differentiated. Thus, this paper also often uses IOF, along with others, interchangeably for the same purpose.

Significant progress on the nutritional knowledge about the post-hatch birds has been generated. Like post-hatch nutrition, pre-hatch nutrition of the chicken embryo is also crucial. Since the incubation period influences embryonic development, hatchability, and post-hatch performance, the number of studies on IOF has risen in recent years.

Typically, a batch of hatch takes around 24–36 h (hatch window) to reach the optimum time to pull all the chickens from the hatcher (8). The sexing, vaccination, and transportation of the chicks from the hatcher to the farm during the hatch window aggravate the stress and debar the chicks from eating and drinking (9). IOF may ameliorate this stress.

Albumen, yolk, and eggshell are the repertoire of the energy and nutrition for the developing chicken embryo. During embryogenesis, different extraembryonic membranes–yolk sac, amnion, chorion, and allantois—play a role in nourishment, protection, respiration, and the storage of metabolites (10). Beneath the eggshell, through a series of developmental processes, a fertile egg transforms into a chick. This process of embryonic development occurs in three major phases—establishment of germ, embryo completion, and emergence (11). Roughly, each phase runs for a one-third period of incubation time. During the first phase, the embryo cannot receive enough oxygen from immature blood cells containing a budding vascular system (12) and compensates for this oxygen deprivation by generating metabolic energy via anaerobic glycolysis of stored glucose within the egg (11). At the second phase, the chorioallantois fully develops and starts balancing O_2_-CO_2_ demand at around Day 8 of embryonic development. This development enhances embryonic growth to reach a structurally complete chicken body around 14 days of incubation (11). The embryo starts thriving on the nutrients conserved within the egg—a rich source of proteins and lipids but a very low amount of carbohydrates (~1% of total nutrients) (10). When deficiency occurs at the second week of incubation, intervention through IOF or IOS helps deflect the effect of nutritional deficiency. During the last week of embryonic development, the embryo gets energy through gluconeogenesis in the liver and yolk sac (10). When yolk sac glycogen depletes, the yolk sac starts maintaining glucose homeostasis and acts as a source of nutrients. With the approach of time toward hatching, after 17 embryonic days (ED), the yolk is resorbed, changing the preference of the delivery route to the air sac and amnion (13).

This review aims to provide an overview of the *in ovo* methods, including the applications of IOT, routes and time of inoculation, the effects of IOT generated by different substances (summarized in Table 1) on embryo and post-hatch poultry, and the challenges and potential of this technique in the poultry industry.

**Table 1:** The effect of *in ovo* feeding of different biological compounds on performance and gut health variables of poultry.

**Bioactive substance**	**Day of injection**	**Target**	**Amount**	**Volume of solution**	**Species**	**Effect** **↓ decrease,** **↑ increase,** **≈ no negative effect or no significant effect**	**References**
Vitamin C and grape seed extract (GSE)	18 ED	Air sac	Vitamin C: 3 mg/egg GSE: 3–6 mg/egg	500 μl	Chicken; Broiler	↑ average daily weight gain, ADFI ≈ feed conversion ratio (FCR) or chick mortality ↓*Coliforms* and *E*. *coli* in the ileum ≈ Ileal population of *Lactobacillus* ↑ Hatchability with IOI of 4.5 mg GSE/egg ↑ Glutathione peroxidase (GPx) activity	(14)
Vitamin C	15 ED	Amnion	Vitamin C: 3 mg or 6 mg/egg	700 μl	Chicken; Ross 308	↑ Increased hatchability with IOI of 6 mg/egg ≈ BWG, FI, FCR ↑ Improved jejunal morphology—increased villus height (VH), villus width (VW), villus height:crypt depth ratio at Day 3 ↓ cholesterol ↑ improved bone strength-tibia resistance and breaking strength at Day 10	(15)
Vitamin C	11 ED	Yolk sac	Vitamin C: 3 mg/egg	100 μl	Chicken; Hy-line Brown	↑ Increased expression of heat shock protein 60, pyruvate dehydrogenase kinase 4, and secreted frizzled related protein 1 at late embryogenic development ↑ Hatchability and plasma vitamin C at hatchlings ↓ Reduced rectal temperature	(16)
Vitamin C	17 ED	Amnion	Vitamin C: 3, 6, 12, 36 mg/egg	100 μl	Chicken; Ross 308	↑ ADG, ADFI, high thigh and leg percentage, and systematic antioxidant capacity at 3–12 mg treatment groups	(17)
Vitamin C	11 ED	Yolk sac	Vitamin C: 3 mg/egg	100 μl	Chicken; Arbor Acres	↑ Total antioxidant content, IgA, IgM	(18)
Vitamin C and glycosaminoglycans	4 ED	Albumen	4 μg of additive (each 100 g additive contained 30 g of chondroitin sulfate, 30 g of glucosamine, 5 g of vitamin C)	100 μl	Chicken; Cobb	↑ Improved the development of bone and cartilages ↑ Ionized calcium in the blood ≈ Macroscopic features (bone weight, bone thickness)	(19)
Vitamin C	18 ED	Air sac	Vitamin C: 3 mg/egg	200 μl	Chicken; Chinese Yellow broiler	↑ Plasma glutathione peroxidase, total antioxidant capacity ↑ Immunity by reducing mRNA expression of IL-1β, IL-6, TNF-α in the spleen	(20)
Vitamin C	At the onset of incubation	Albumen	Vitamin C: 0, 2, 4, 6 μg	100 μl	Chicken; Cobb	↑ Stimulate egg bursal development and lymphocytosis ≈ No difference in the total leukocyte count	(21)
Vitamin C	At the onset of incubation	Albumen	Vitamin C: 6 μg	100 μl	Chicken; Cobb	↑ Ionized calcium in the plasma Act as a long-term stimulator and modulator of the immune system	(22)
Vitamin C	At the onset of incubation	Albumen	Vitamin C: 6 μg	100 μl	Chicken; Cobb	IOI did not ameliorate the negative effect of high rearing temperature on production performance	(23)
Vitamin D	18 ED	Amnion	Vitamin D3 and 25-hydroxycholecalciferol [25(OH)D3]: 0.6–2.4 μg D3/25(OH)D3, or combination of both	50 μl	Chicken; Ross 708	↑ Serum 25(OH)D3, when IOF was given at ≥1.2 μg of D3, or ≥1.2 μg 25(OH)D3 ≈ No negative effect on broiler hatchability index or chick quality	(24)
Vitamin D	18 ED	Amnion	0.2, 0.6, 1.8, 5.4 μg 25(OH)D3 with commercial diluent	100 μl	Chicken; Ross 708	↑ Bone breaking strength (BBS) in male birds on Day 28 posthatch, but no change in female birds ≈ No positive effect on the bone development and strength through Day 28 post hatch Commercial vaccine diluent or the injection process had adverse effect on BBS	(25)
Vitamin D	17 ED	Allantoic cavity	0.625, 1.250, or 1.875 μg 25(OH)D3	300 μl	Chicken; Cobb broiler	Decrease mean hatching time (4–5 h earlier) ≈ No negative effect on hatching or neonate qualities	(26)
Vitamin D and minerals	17 ED	Amnion	Organic minerals, phosphate, and 240 IU of vitamin D3	600 μl	Chicken; Cobb 500	IOF of minerals induces higher mineral uptake from the yolk ↑ Whole-bone stiffness in the hatchlings and on Day 38 ↑ Ash content of the bones on Day 38	(27)
Cysteine and methionine	17.5 ED	Amnion	L-Meth 5.9 mg, L-Cys 3.4 mg	1,000 μl	Chicken; Ross 308	↓ HSP70 ↑ GSH-Px ↓ Corticosterone ↑ T-SOD, Cu-Zn-SOD of serum, SI, liver, pectoral muscle ↓ MDA (Malondialdehyde) ≈ CAT (Catalase) ↓ Triglycerides, total cholesterol, VLDL, HDL ↓ embryonic mortality, but not significant	(28)
Beta-hydroxy-beta-methylbutyrate (HMB)	7 ED	Air sac	1,000 μg	1,000 μl	Chicken; Arbor Acre	↑ Hatchability by 4.34%, body weight, average daily body weight gain, pectoral muscle percentage ↑ Plasma growth hormone, insulin, and insulin-like growth factor-1 ↑ mRNA expression of myogenic transcription factors, myogenic differentiation, and myogenin 7 ED air cell injection is beneficial than 18 ED amnion injection of HMB	(29)
HMB, arginine, and egg white protein (EWP)	21 ED/23 ED	Amnion	1,500 μg of HMB	1,500 μl	Turkey	↑ Two- to three-fold increase in jejunal sucrase, maltase, and leucine amiopeptidase activities in HMB and arginine IOF at Day 14 ↑ Glucose uptake in the embryo in IOF of HMB and EWP ↑ overall increase in jejunal nutrient uptake and digestion	(30)
HMB and carbohydrates	17.5 ED	Amnion	HMB: 1,000 μg Carbohydrate solution: 25 g of maltose/L, 25 g of sucrose/L, 200 g of dextrin/L	1,000 μl	Chicken; Ross	↑ Villus width and surface area (45%) ↑ Intestinal capacity to digest disaccharides by increasing jejunal sucrase-isomaltase activity and maltase activity ↑ Body weight of hatchlings, and Day 10 chicks	(31)
HMB and carbohydrates	17.5 ED	Amnion	HMB: 1,000 μg Carbohydrate solution: 25 g of maltose/L, 25 g of sucrose/L, 200 g of dextrin/L	1,000 μl	Chicken; Cobb 500 and Ross 308	↑ Hatchling weights (5–6%) ↑ Body weight until Day 25 ↑ Liver glycogen (two-to five-fold) ↑ Breast muscle (6 to 8%)	(4)
HMB and dextrin	18 ED	Amnion	10% dextrin, 0.4% Ca–HMB in 0.4% NaCl	600 μl	Chicken; Cobb 500	↑ Glycogen reserves in liver and pectoral muscle on 19 and 20 ED and at hatch ↑ BW, ADFI, ADG, and FCR ↑ Myoblast proliferation on 19 ED and Day 5 ↑ Myofiber diameters, pectoral muscle weight (PMW) and PMW-to-BW ratio on Day 35	(32)
Betaine	Before incubation	Yolk sac	Betaine: 2.5 mg, before incubation CORT: Postnatal day 7, subcutaneous injection	100 μl	Chicken; Rugao yellow	↓ CORT-induced cholesterol deposition ↑ hepatic expression of cholesterol biosynthesis genes and ACAT1 protein Prevent CORT induced down regulation of LXR and CYP27A1in liver ↑ CpG methylation on the promoter regions of LXR and CYP27A1	(33)
Betaine and choline	12 ED	Air sac	Betaine: 0.25, 0.375, 0.50 mg Choline: 0.25, 0.375, 0.50 mg	500 μl	Chicken; Ross 308	↑ Hatching weight, final BW ↓ FCR and abdominal fat percentage ≈ Carcass yield, breast muscle, leg and wings percentage ≈ IgM, IgG, and total antibody titers (IgT)	(34)
Carbohydrates	14 ED	Amnion/yolk sac	Either 50 mg of glucose/fructose/ribose	500 μl	Chicken; Cross of Cornish and Plymouth Rock	↑ Expression of humoral immune-related genes (IL-6 and IL-10), chicken growth hormone, and IGF-II during late-term embryonic and early post hatch days in case of IOF of glucose ↑ Expression of IGF-II, IL-2, IL-12, IFN-gamma, and mucin gene in fructose and ribose supplemented chickens	(35)
Carbohydrate	21 ED, 25 ED	21 ED: Yolk sac/allantoic cavity25 ED: Amnion/yolk sac	1 ml of 10% glucose	1,000 μl	Turkey	↑ Humoral immune response on both 21 and 25 ED groups ≈ Cell-mediated immune response ↓ Hatchability ↓ Liver weight ↑ Bursa weight ↑ BW in IOF of glucose through yolk sac route	(36)
Carbohydrates	18.5 ED	Amnion	250 mg of glucose, fructose, sucrose, maltose, or dextrin in 1 ml of diluent	100–1,000 μl	Chicken; Ross 708	Hatchability decreased with an increase of injection volume >400 μl solution of fructose and sucrose, decreases hatchability >700 μl solution of glucose, maltose, or dextrin decreases hatchability ↑ BW of hatchlings and BW were positively related to increase of the amount of carbohydrate	(37)
Carbohydrates	14.5 ED	Amnion	1.5% maltose and 1.5% sucrose; or 2.5% maltose and 2.5% sucrose; 3.5% maltose and 3.5% sucrose; or 4.5% maltose and 4.5% sucrose	200 μl	Pigeon	A low level of carbohydrates (1.5 to 2.5% maltose and sucrose) increased hatchability A high level of carbohydrates (4.5% maltose and sucrose) decreased hatchability ≈ Liver glycogen reserve ↑ BW, pectoral muscle weight (PMW), pectoral muscle glycogen reserve at hatch, yolk sac nutrient utilization, enteric development	(38)
Carbohydrate	2 or 4 ED	Air sac	0.3–2g glucose/kg of egg weight	–	Chicken; White Leghorn	↑ Limb defects/heart defect in IOF of higher amount of glucose ↑ Disrupted cell proliferation and apoptosis in high glucose injection	(39)
Prebiotics	12.5 ED	Amnion	Chitooligosaccharide (COS) (5 to 20 mg) and chlorella polysaccharide (CPS) (5 to 20 mg)	500 μl	Chicken; Cobb 500	↑ Increased the population of *Lactobacillus johnsonii, Bacteroides coprocola*, and *Bacteroides salanitronis* in COS group ↓ Decreased the population of opportunistic pathogenic bacteria ↑ Increased gluconeogenesis, L-isoleucine degradation, L-histidine biosynthesis, and fatty acid biosynthesis in COS group Response to prebiotics increased with age	(40)
Prebiotics	12 ED	Air sac	Galactooligosachharide 3.5 mg	200 μl	Chicken; Ross 308	↑ Increased the relative abundance of Bifidobacterium in the cecum ↓ Decreased the relative abundance of Lactobacillus in the ileum ↑ Upregulated the expression of cytokine genes (IL-1β, IL-10, and IL-12p40) and MUC6 in the jejunum and cecum ↑ Upregulated the expression of host defense peptides (AvBD1 and CATHL2) in the cecum ↑ Upregulated the free fatty acid receptors (FFAR2 and FFAR4) in the intestine	(41)
Prebiotics	12 ED	Air sac	Galactooligosachharide 3.5 mg	200 μl	Chicken; Hubbard	↓ Reduced the heat-induce Th2 immune responses (downregulation of IL-4) ↑ Downregulated the expression of the cytokine genes (IL-10 and IL-1p40) ↓ Reduced oxidative stress responses by downregulating the expression of CAT and SOD genes in heat stress	(42)
Prebiotics	12 ED	Air sac	Galactooligosachharide 3.5 mg	200 μl	Chicken; Ross 308	↑ Increased BW (day 42), ADFI, and FCR (at finisher phase) ↓ Reduced foot-pad dermatitis and negative effects of HS ≈ No effect on hatchability	(43)
Prebiotics	12 ED	Air sac	1.9 mg of Raffinose family oligosaccharides (RFOs)	200μl	Chicken; Cobb, Ross, and Hubbard	↓ Hatchability ↑ BW	(44)
Prebiotics	12 ED	Air sac	0, 1.5, 3.0, and 4.5 mg RFOs	200μl	Chicken; Cobb 500	↑ Increased the villus height and villus height:crypt depth ratio in ileum by high dose ↑ Upregulated the expression of T cell and B cell gene markers (CD3 and chB6) in ileum by high dose	(45)
Prebiotics	12 ED	Air sac	1.9 mg of RFOs	200μl	Chicken; Ross 308	↑ Improved the blood lipid profile ↑ Increased villi surface ↑ Improved the gut bacterial composition through reducing the population of Clostridia and coccidia	(46)
Probiotics	17 ED	Yolk sac	5 × 10^9^ and 1 × 10^7^ cfu *B. bifidum* ATTC 29521; and of 5 × 10^9^ and 1 × 10^7^ cfu *B. longum* ATTC 15707	200 μl	Chicken; Cobb 500	↑ Improved BWG and FCR ↑ Ileal villus height and villus height/crypt depth ratio ≈ Insignificant change in ileal crypt depth	(47)
Probiotics	19 ED	Amnion	Lactic acid bacteria containing 10 strains @ 7 log cfu Challenge: 4.5 × 10^4^ cfu/ ml *E. coli*	200 μl	Chicken; Ross 308	↑ BWG ↑ Lactic acid bacteria at hatchlings ↓ Enterobacteriaceae colonization at *E. coli* challenged birds at hatchlings and at 7-day-old chickens ↓ Reduced mortality in the challenged chickens at Day 7 ≈ hatchability	(48)
Probiotics	18 ED	Amnion	10^4^ cfu of FloraMax^®^-B11 (*Lactobacillus salivarius* and *Pediococcus parvulus*) Challenge: with virulent MDV (vMDV; strain 583A)	–	Chicken; White Leghorn 15I_5_x7_1_ broiler	≈ Insignificant difference in protection against vMDV ≈ Hatchability ↓ Lactose positive Gram-positive bacteria ↑ BW, surface area of ileal villi, protection against SE incidence	(49)
Probiotics	18 ED	Amnion	10 × 10^2^ cells of *Citrobacter freundii* 97A11, or *Citrobacter spp*. 97A4, or a mixture of *L. salivarius* and *Pediococcus spp*	200 μl	Chicken Ross 708 broiler	↑ Antioxidant capacity ↓ Inflammatory status Pioneer colonizers showed differences in proteomic profile at hatch day which are related to immune and skeletal muscle development ↑ Gluconeogenesis in Lactobacillus treatment group	(50)
Probiotics	18 ED	Amnion	10 × 10^2^ cells of *Citrobacter freundii* 97A11, or *Citrobacter spp*. 97A4, or LAB (a mixture of *L. salivarius* and *Pediococcus spp*)	200 μl	Chicken Ross 708 broiler	↑ LAB-treated group showed enhanced immune response function and skeletal development ↑ LAB increased colonization of butyrate-producing bacteria (by 3 and 10 days) and segmented filamentous bacteria (by 10 days) ↓ LAB decreased *Enterococcaceae*	(51)
Probiotics	18 ED	Amnion	2 strains of *Bacillus amyloliquefaciens* and one strain of *Bacillus subtilis*; 5 × 10^7^ cfu/ml	200 μl	Chicken; Ross 308	↓ Reduced Gram-negative bacteria on Day 0 and Day 7 post hatch↑ BW and BWG (on Day 0 to Day 7 post hatch)	(52)
Probiotics	17.5 ED	Amnion	Seven probiotic treatments in various cfu including *E. faecium, B. subtilis* (dose up to 10^9^ cfu/egg)	500 μl	Chicken; Ross 308	↓ Hatchability (by 10%) due to injection ↓ Reduced number of SE positive chicks in SE challenged chickens	(53)
Probiotics	18 ED	Amnion	Multi-strain Lactobacilli mixture (*Lactobacillus salivarius, Lactobacillus reuteri, Lactobacillus crispatus*, and *Lactobacillus johnsonii*); at three doses−10^5^, 10^6^, 10^7^ cfu/egg	100 μl	Commercial broiler	↑ Splenic expression of cytokines (IFN-α, IFN-β, IFN-γ, IL-8, and IL-12) ↓ IL-2, 6, 8 in cecal tonsils ≈ No significant difference in bursa, except upregulation of IL-3 in high doses ↑ Increased serum IgG and IgM responses in keyhole limpet hemocyanin immunized birds	(54)
Probiotics	18 ED	Amnion	*Lactobacillus animalis* 10^6^cfu and *Enterococcus faecium* 10^6^ cfu	50 μl	Chicken; Ross 708	≈ No differences in hatch parameters, BWG, and mortality ≈ No significant difference in FCR ≈ Did not impact hatch parameters	(55)
Probiotics	18 ED	Amnion	*Lactobacillus acidophilus, Bacillus subtilis*, or *Bifidobacterium animalis* (10^3^-10^6^ cfu)	50 μl	Chicken	↓ Decreased hatchability, BW in IOF of *Bacillus subtilis* ≈ Did not impact hatchability in IOF of *L. acidophilus* and *B. animalis*	(56)
Synbiotics	12 ED	Air sac	Synbiotic: *Lactobacillus salivarius* + GOS: Synbiotic: *L. plantarum* + RFO	200 μl	Chicken; Cobb 500FF	Synbiotic (*L. plantarum* + RFO) significantly changed DNA methylation of metabolic genes (downregulated ANGPTL4 and upregulated NR4A3) in liver. Lactobacillus synbiotics increased BW, did not affect FCR, and did not change immune related genes (SYK and KLHL6) in liver	(57)
Hormone: corticotropin-releasing hormone (CRH)	10–18 ED	Albumen/air cell/amnion	0.1, 1, or 2 μg of CRH	100 μl	Chicken; Cobb 500	≈ No substantial effect on hatching time and hatchability	(58)
Hormone: thyrotropin-releasing hormone	24 ED	Air cell/bottom of the egg	0.1–5 μg	200 μl	Turkey	↑ Embryonic blood plasma T_4_ (by four-fold) ↑ Embryonic blood plasma T_3_ only with high dose through bottom of the egg	(59)
Hormone: corticosterone	11 ED	Yolk sac	0.2 or 1 μg	100 μl	Chicken	↓ The post-hatch growth rate ↑ Aggressive behavior and plasma corticosterone ↓ Hypothalamic expression of arginine vasotocin and CRH	(60)
Hormone: thyroxine	18 ED	Amnion	65 ng	500 μl	Chicken	↓ Second-grade chicks, yolk sac weight at hatch ↑ Body weight at hatch ↓ Incidence of ascites and mortality due to ascites	(61)
CpG-ODN, polyinosinic:polycytidylic acid (Poly I:C), and cyclic polyphosphazene (CPZ)	18 ED	Yolk sac	Trial A: CpG ODN @ 50 μg; Poly I:C @ 2.5 μg; cyclic polyphosphazene (CPZ)75B @ 10 μg; Loxoribine @ 2.5 μg Trial B: CpG-ODN @ 50 μg Poly I:C @ 5 μg	100 μl	Chicken	From Trial A: Birds challenged with yolk sac infection showed 80, 65, and 60% survival with IOI of CpG-ODN, poly I:C, and CPZ, respectively; and reduced early chick mortality. CpG-ODN also improved the clinical scores From Trial B: The lower dosage (2.5 μg/egg) of CPZ is as potent as higher dosage (5 μg/egg)	(62)
CpG-ODN	18 ED	Amnion	50 μg of carbon nanotubes-CpG-ODN 50 μg of lipid surfactant-CpG-ODN 50 μg of unformulated CpG-ODN Saline	100 μl	Chicken	↑ Survival rate in the *E. coli* changed chicken (saline treatment: 20 to 30% CpG-ODN formulations: 60 to 80%) ↓ Lower bacterial loads and clinical scores in the formulated CpG-ODN treated groups compared to the unformulated CpG-ODN or saline groups CpG-ODN formulated with carbon nanotubes and lipid surfactant showed an immunomodulatory effect against early infection with *E. coli* in broilers	(63)
CpG-ODN (class-B)	18 ED	Chorioallantoic sac	50 μg CpG-ODN	50 μl	Chicken	↑ Increased expression of IFN-c, IL-1b, IL-6, and IL-8, and oligoadenyl synthetase A mRNA ↓ Reduced infectious bronchitis virus propagation in different embryonic tissues in the IBV challenged embryos	(64)
CpG-ODN	18 ED	Amnion	25 μg of CpG-ODN	100 μl	Chicken	↓ Reduced *Salmonella entiritidis* colonization by <10-fold by stimulating immune responsiveness of heterophils	(65)
CpG-ODN	18 ED	Amnion	25 μg of CpG-ODN	100 μl	Chicken	↑ Increased survival rate by two-fold ↓ Decreased bacterial loads and pathology in the *Salmonella typhimurium* challenged chickens Immuno-protective against intracellular bacterial infection of *Salmonella typhimurium*	(66)
CpG-ODN	18 ED	Amnion	50 μg CpG-ODN	100 μl	Chicken	↑ Survival rate in *E. coli* infection ↑ Immuno-protective effect against *E. coli* with IOI of polyphosphazene formulated CpG-ODN	(67)
CpG-ODN	18 ED	Amnion	25 μg CpG-ODN	100 μl	Chicken	Encapsulated CpG moderately improved the herpes virus of turkey (HVT) vaccine efficacy against Marek's disease and reduced tumor incidence from 53 to 33%	(68)
CpG-ODN	18 ED	Amnion	50 μg of CpG-ODN	100 μl	Chicken	In *E. coli* challenged chicks: ↓ reduced clinical scores in CpG-ODN treated chicks ↑ Upregulation of proinflammatory cytokines [IL-1, IL-4, IL-6, IL-10, IL-18, IFN-γ, IFN-α, and lipopolysaccharide induced tumor necrosis factor (LITAF)] in spleen and lungs ↑ Significant enrichment of macrophages, CD4+ and CD8+ T-cell subsets in both spleen and lungs ↑ Upregulation of CD40 but not CD86 ↑ CD4 and CD8 expression ↑ Protection in neonatal chicks against *E. coli* infection	(69)

## Applications of *in ovo* Technology

### *In ovo* Stimulation

Earlier injections into the air sac with prebiotics and synbiotics are aimed to modulate gut microflora and are called *in ovo* stimulation. Bioactive compounds are delivered to the early-stage embryos (up to 12 ED) through *in ovo* injection because they can stimulate the growth of microbes and can positively affect intestinal development and health (41). The chorioallantoic membrane (CAM) is highly vascularized at this stage. Thus, the prebiotics (small-weight oligosaccharides) can pass from the air sac to the blood vessels surrounding the embryo via passive transfer. The *in ovo* stimulation with prebiotics enhances the growth of indigenous microflora in the egg, leading to the growth of the microbiome with the embryo's development (41).

### *In ovo* Feeding

**Figure 1:**
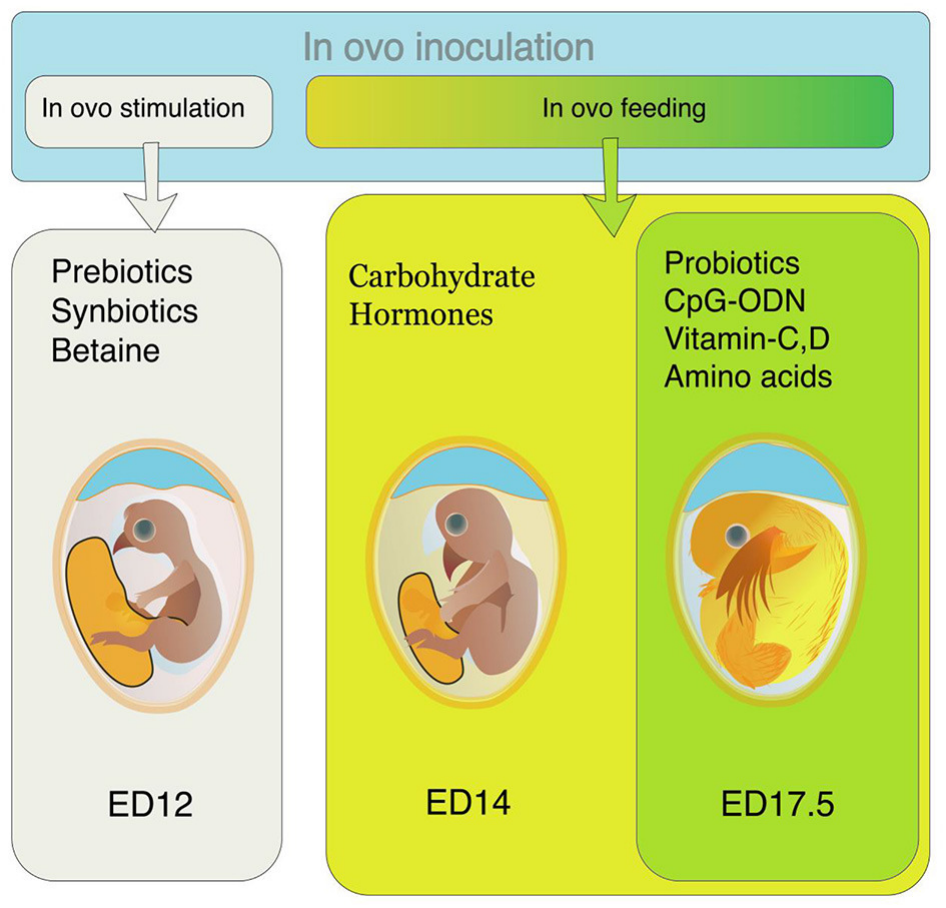
Preferred time of *in ovo* inoculation for different bioactive compounds. For *in ovo* stimulation, supplements like prebiotics, synbiotics, and betaine are inoculated on ED 12. For *in ovo* feeding, the preferable time of supplementation is either ED14 (for carbohydrates, hormones, and alike compounds) or ED 17.5 (for probiotics, CpG-ODN, vitamins, amino acids, etc.).

Different nutrients are supplemented through amnion from ED 14 to ED 18. This supplementation is called *in ovo* feeding (Figure 1). This injection provides nutritional support to the pre- and post-hatch chicks (4). The IOF was initially intended to provide the nutrients required by the embryos and support the post-hatch chicks during the hatch window, but this technology is being used for supplementing different bioactive compounds in recent years (5).

### *In ovo* Sexing

Sexing via IOT can contribute to a regime shift regarding animal welfare and ethical concerns by avoiding killing day-old male chicks in commercial layer production. This gender determination is of critical importance in broilers as well. The separation of males and females facilitates better management practices as their growth and maturation patterns differ (70). Some invasive and non-invasive methods are used for *in ovo* sexing. Invasive methods include quantifying DNA by spectroscopic assays (71) and measuring the concentration of estrogen sulfate (72) in the embryonic tissues. Among non-invasive methods, virus spectroscopy of eggshells (73), fluorescence spectroscopy of embryonic blood can be done within ED 3 to ED 4 (74), and hyperspectral imaging of down feathers around 14 ED (75). A non-invasive method is preferable as it can ward off the contamination of the embryo and the adverse effects on embryo development and hatchability. A non-invasive method consists of an electromagnetic radiation transmitter and detectors. This method can analyze the volatile organic compounds (a unique combination of compounds released by the chicken egg, which varies with male-female and fertile-infertile eggs) in the egg's air cell to determine the gender and fertility as early as Day 1 of incubation (70).

### *In ovo* Chick Model for Epigenetic Research

The IOT has the potential to use chicken embryos as a powerful model for epigenetic research. The use of chicken embryos for epigenetic studies surpasses the benefits of mammalian models because the chicken embryo develops fast, the blastoderm is easily accessible, and the embryo grows external to the body (7).

### *In ovo* Stress Model

The IOT can be used to understand the effect of the stressor during embryogenesis on the development of genotypic traits of chickens and other parameters—hatchability, late embryonic mortality, body weight, etc. In a study, the researcher injected egg with corticosterone at the rate of 10 ng/mL (corticosterone was diluted in 50–60 μl of sesame oil) of egg contents to simulate the stress in the embryos and compared the effects against different factors (maternal stress, strains of hen, sesame oil treatment) (76). The induced *in ovo* stress affected the sex ratio, late embryonic mortality, hatchability, and body weight at hatch and post-hatch periods.

### *In ovo* Chick Model for Human Medicine

The chicken embryo model is a cheaper, faster, and reproducible technique that can be used to study the effect of different compounds during embryogenesis (77). Unlike other vertebrate animal models, the mother would not be affected or killed due to the adverse effect of the compounds of the investigation. This embryo has been used as a tool for *in ovo* electroporation (a precise delivery of genetic material) to study the developmental events, the effects of gene activation, or overexpression on downstream transcriptional regulation (77, 78).

The chicken embryo models have been used in the study of teratogenicity (79), disease development (39, 80), cell migration and histogenesis, and causal mechanisms of neurocrystopathies (abnormal specification, migration, differentiation, or death of neural crest cells during embryonic development) (81). *In ovo* transplantation of neural crest cells (NC) derived from keratinocyte cells (KC) showed that these NC-KC cells could migrate to the neural crest region, grow within the egg, and produce different NC derivates, which is the indication of maintaining their pluripotent state (82).

Despite many developments in the use of the *in ovo* chick model, this model cannot serve as a complete surrogate of human development because of the absence of protective mechanisms which are ensured by the fetus in the uterus (83).

## Routes and Time of *in ovo* Delivery

**Figure 2:**
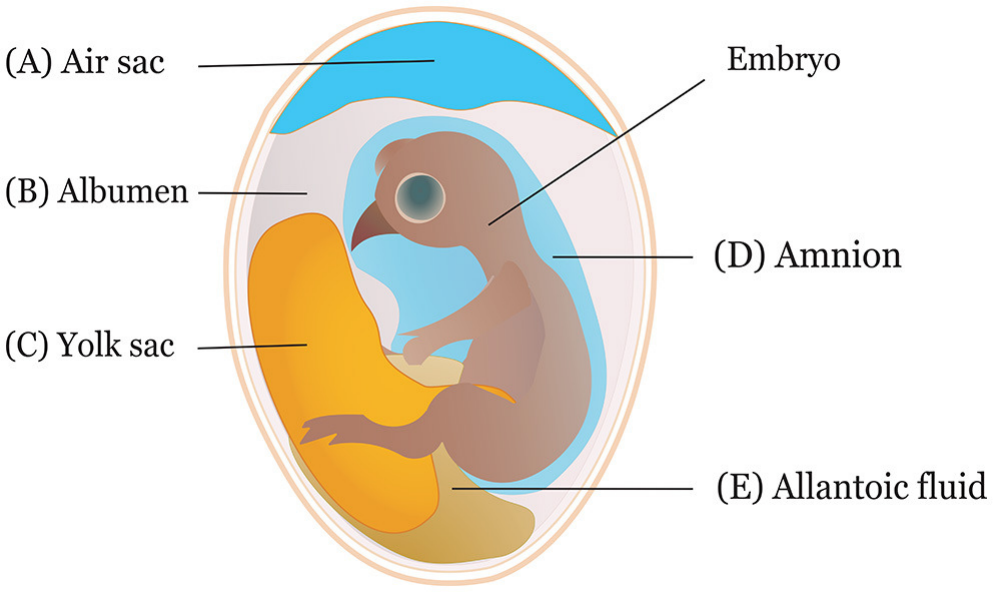
Sites of *in ovo* inoculation. Eggs can be inoculated through **(A)** air sac, **(B)** albumen, **(C)** yolk sac, **(D)** amnion, and **(E)** allantoic fluid.

It is crucial to consider the route and time of *in ovo* delivery of the compounds as it may affect the output of the intervention. Researchers use different routes for *in ovo* inoculation (Figure 2) to investigate the effects of various bioactive compounds. The IOI of amino acid into the eggs showed less hatchability when injected through the CAM or amniotic cavity than the eggs injected into the yolk sac or extraembryonic cavity (84). Typically, an air sac is targeted when the injection is made in the early phase of the incubation (45, 85). Amnion is targeted to deliver compounds in the amniotic fluid during the later phase of the incubation when the neonatal chicks inside the egg consume the amniotic fluids (29). The selection of the route depends on the type of biological compounds to be delivered. For probiotics administration, the efficiency of IOD into the air sac was compared with oral gavage, spray, and vent lip methods, where IOD showed a reduced efficiency in harnessing benefits and reducing Salmonella colonization in the challenged chickens (86). A bioluminescent non-pathogenic *E. coli* DH5α was injected into either the amnion or air cell regions of the embryos at ED 18 to measure bacterial load in different visceral tissues (87). The study suggested IOD into the amnion yields a higher bacterial concentration in the tissues, notably in the ileum and ceca, than injection into the air sac.

The compounds are tested through different “trial and error” experiments to find a successful route of administration or qualify a compound for IOD. For example, corticotropin-releasing hormone (CRH) was injected into the air cell, albumen, and amniotic fluid during incubation, and the plasma level for the injected compound when assessed showed no difference than the non-injected control (58). The result disqualified the compound CRH for IOD as this failed to bring beneficial outcomes.

Nutrients are absorbed via different transporters. The abundance of the transporters and the pattern of mRNA expression of the transporters could dictate the choice of routes. Such as, the yolk sac translates a higher amount of sodium-dependent vitamin C transporter 1 (SVCT1) than amnion and is considered the optimum route for vitamin C inoculation (88).

The time of injection is determined by the type of compounds and the desired output to bring in the embryos. As HMB helps in forming the first generation of myoblasts and this formation occurs around 7 ED, injection of HMB into air cell on ED 7 showed more beneficial effect than injection into embryonic amnion on ED 18 (29).

## Tools for *in ovo* Delivery

Most of the researchers are using manual tools for *in ovo* delivery. These manual tools are time-consuming and often a limiting factor to scale up the sample size of research. However, with increasing demand from the breeder and hatchery, some companies manufacture IOD machines with efficiency. The machines can deliver biological compounds to eggs at the rate of 12,000–70,000 per hour, namely, Sanovo vax^®^ automatic (62,000/h), Vinovo inject^®^ flex (Liver embryo detection), Egginject^®^ (60,000/h), Embrex Inovoject (70,000/h), and Embrex Inovoject (12,000–20,000/h). The machines have beneficial features, for example, automatic needle sanitation, dual needle system, vaccine saving technology, adjustable needle depth, candling technology, live embryo detection, and viable egg transfer.

*In ovo* injection of vaccines enormously reduces the time needed for vaccinating eggs in a large-scale hatchery and labor cost; increases the accuracy of vaccination and immunity; without significantly hampering the hatchability. But IOV may show some discrepancies depending on the route, embryonic growth, *in ovo* machines, and maneuvers related to vaccine production and aseptic delivery (89).

## *In ovo* Delivered Bioactive Compounds and Their Effects

### Vitamins

L-ascorbic acid ameliorates the stress-related responses when birds go through stress (90) due to the exposure of overheat during the end of the incubation (91).

IOF of ascorbic acid increased hatchability (14, 15, 18) but did not affect feed intake and growth parameters during the starter phase (15). However, it improved jejunal morphology (increased villus height, villus width, villus height: crypt depth ratio) and decreased cholesterol (15). In another study, IOF of ascorbic acid increased post-hatch growth, improved leg muscle development, and increased plasma antioxidant level in broilers (17). Zhang et al. (17) also reported that IOF of 3 and 6 mg ascorbic acid increased body weight (BW), average daily gain (ADG), and average daily feed intake (ADFI) during the grower phase (Day 14–28), but a high dose (36 mg) negatively impacted growth performance, and 12 mg beneficially decreased plasma Malondialdehyde at the grower and finisher phase of a bird's life (17). IOF of vitamin C increased plasma level of vitamin C in newly hatched chicks, decreased embryonic mortality, and increased hatchability (18, 88).

IOF of vitamin C resulted in the improved bone characteristics (tibia resistance and breaking strength) at post-hatch (15, 19) and helped in the development of cartilages and bones by improving bone mineral content and bone mineral density (19). Santos et al. (19) evaluated the effect at Day 43 and reported that IOF of broiler eggs with 4 μg of additive (30% chondroitin sulfate, 30% glucosamine, 5% vitamin C) in 100 μl water and addition of these nutrients in feed did not change the femur cartilage macroscopy, but synergistically improve the femur cartilage weight.

The IOF of vitamin C reduced the stress status of the spleen by regulating the expression of inflammatory cytokines at post-hatch Day 42 (18). Also, the IOF enhanced humoral immunity with increased immunoglobulin production and lysozyme activity at post-hatch Day 21, reflecting enhanced humoral immunity. Another study by El-Senousey et al. (20) also showed that IOF of 3 mg ascorbic acid through the air sac at ED 18 in the eggs of Chinese yellow broiler chicken improved immunity and increased the antioxidant production at Day 1. The immunological improvement was evident by a decrease in the mRNA level of pro-inflammatory cytokines (IL-1β, IL-6, and TNF-α) in the spleen. Also, the injection up-regulated the expression of antioxidant genes [Glutathione peroxidase (GSH-PX) and superoxide dismutase (SOD)]. The authors suggested that the reduced expression of the immune-related genes might be due to the production of antioxidants and the consecutive elimination of the reactive oxygen species by the antioxidants.

Vitamin C can increase DNA demethylation and histone demethylation, and influence the epigenome of the liver through regulating gene expression (16). Zhu et al. (16) found that IOF of vitamin C at ED 11 increased the gene expression of heat shock protein 60 (HSP60), pyruvate dehydrogenase kinase (PDK4), and secreted frizzled-related protein 1 (SFRP1) to protect the embryo against heat stress. But other studies did not show a similar epigenetic adaptation in combating embryonic heat stress (22, 23).

Vitamin D has two forms—vitamin D2 and vitamin D3. Vitamin D_3_, present in animal food, is more efficient to absorb than vitamin D_2_ of plant origin. But feeding broiler breeders with animal byproducts may increase the transfer of microorganisms to eggs during egg formation in the oviduct. So, the eggs might not get sufficient vitamin D3. But IOI of vitamin D_3_ may resolve the problem of microbial transfer.

Again, vitamin D increases calcium (Ca) absorption from the gut by genetic manipulation in enterocytes, increasing the production of Ca_2_+ transporter, calbindin D28K, vitamin D receptor, ATP-dependent Ca_2_+ pump and, thus, increasing calcium uptake (92). In the kidney, it increases Ca reabsorption and in bone, it increases osteoclasts formations and bone resorption. Understanding of embryogenic metabolism of vitamin D will determine the significance of IOF of vitamin D.

The CAM develops within 9 to 14 d of incubation, but this vascularized membrane first contacts the eggshell between 9 and 12 d of incubation. After the formation of CAM, the embryo shifts its source for calcium from yolk to eggshell. When the villus cavity expresses carbonic anhydrase to dissolute calcite from eggshells; and capillary-covering cells are fully differentiated, CAM starts to transfer calcium from eggshells (93). This transfer of Ca directly manifests in the mineralization of the embryo skeleton.

The Ca usage from the eggshell and Ca transport through CAM depends on vitamin D (94). Vitamin D deficiency in eggs may result in Ca deficiency, failure to achieve pipping pre-hatching position, which is required for the transition to pulmonary respiration, and develop hypoxia; and increased the incidence of late embryonic death (94). Reduced Ca dissolution from eggshells may end up with higher shell thickness. The embryo from an egg with less shell thickness has higher shell breaking strength, goes through successful pipping, and achieves higher hatchability (95).

IOI of 1.2 μg of vitamin D_3_, or a more active form of vitamin D_3_ [25(OH)D_3_] increases serum level of 25(OH)D_3_ in the broiler embryos of Day 19.25, but this injection does not affect hatchability and hatchling BW (24). But a later study by Fatemi et al. showed that IOI of a larger dose (2.4 μg) of 25(OH)D_3_ at Day 18 had increased the breast meat yield and BW by reducing the inflammatory response indicator (plasma alpha-1-acid glycoprotein) at post-hatch age (96). Vitamin D_3_ has a shorter half-life of 12–24 h, but 25(OH)D_3_ has 2–3 weeks (97, 98). So, 25(OH)D_3_ in the blood is available for later use and can be converted to calcitriol, an active form of vitamin D (24). IOI of 25(OH)D_3_ can be used to increase the serum concentration of vitamin D.

Bello et al. conducted a study by IOI of 0.2, 0.6, 1.8, or 5.4 μg of 25(OH)D_3_ at ED 18 in different treatment groups (25). Bone-breaking strength (BBS) was elevated due to increased bone Ca concentration in males on post-hatch Day 28 in all groups except embryos injected with 5.4 μg of 25(OH)D_3_, but females did not get any benefit regarding BBS. The authors linked this divergence of the effects to the sex hormone variation. The results of BBS from 5.4 μg dose did not conform to results from other doses, and this non-conformity was attributed to a toxic effect of 25(OH)D_3_ by the authors. In addition, they claimed that a single IOF of 25(OH)D_3_ cannot improve bone mineral density and percent residual bone ash; but can induce a right shift in bone mineral profile to achieve favorable Ca concentration. This gap of not achieving bone mineral density/stiffness and bone mineralization was fulfilled by IOF with a combination of organic and inorganic minerals and 25(OH)D_3_ (27).

### Amino Acids and Their Metabolites

Heat stress (HS) generates reactive species. This HS causes oxidative stress that may lead to upregulation of the defense system to adapt the cells against the stress, lead to cell injury [oxidation of lipids, DNA, protein, etc. within the cell by reactive oxygen species (ROS), ionic imbalance, or activation of protease], and cell death (by apoptosis or necrosis) (99, 100). Cells respond to HS by producing heat shock protein 70 (HSP70), corticosterone (CORT) hormone to protect the cell. Methionine and cysteine play significant roles with their metabolites, such as S-adenosylmethionine (SAM), polyamines, taurine, and glutathione. Their presence is crucial also for protein synthesis, metabolic reactions, immune functions, and oxidation activities (101).

A combination of L-methionine and L-cysteine (5.9 and 3.4 mg, respectively), was injected at ED 17.5 in eggs which were incubated under heat stress (39.6°C for 6 h/d) from ED 10 to ED 18 (28, 102). This combination reduced the level of heat shock protein-90. IOI improved the values of total antioxidants capacity, GSH and GSH/GSSG in tissues, upregulated the expression of IGF-I and TLR4, 29% increase in villus area, reduced CORT concentrations, and lipid profile of hatched chicks. A significantly lower level of lipid and CORT indicates adaptation to HS.

Dietary supplementation of beta-hydroxy-beta-methylbutyrate (HMB), a leucine metabolite, can act as a precursor of beta-hydroxy-betamethylglutaryl coenzyme-A (HMG-CoA) and contributes to cholesterol synthesis, which is required for maximal cell functions and cell growth (29, 103). It decreases chicken mortality, increases carcass yield, and improves protein metabolism in muscle in case of attenuating proteolysis (104). A study by Tako et al. (31) showed that IOF of 1,000 μg of HMB on ED 17.5 through amnion enhanced the enterocyte proliferation and differentiation and on ED 20, reduced protein degradation, increased villus surface area at Day 3, and elevated BW at Day 10. In turkey, IOF of a combination of HMB, arginine, and egg white protein at ED 23 improved jejunal uptake of glucose and alanine during the post-hatch period and improved growth performance (30).

The IOF of HMB in the broiler eggs at ED 7 or ED 18 showed that the exogenous HMB could compensate for the metabolic deficiency of HMB in the embryo (29). This study showed that IOF of HMB could improve hatchability and increase the broiler's breast muscle size and BW in the post-hatch period. The authors suggested that the muscle hypertrophy was due to the stimulation of muscle protein, enhanced mitotic activity of satellite cells with upregulation of myogenic transcription factors-MyoD and myogenin, activation of GH-IGF-1 axis, and cholesterol synthesis by HMB. This cell proliferation can recover the muscle cells in the late embryonic stage when increased energy demand is met by producing glucose from muscle through gluconeogenesis (4).

Betaine, a metabolite of choline, donates a methyl group during the methylation process of DNA (105). Transcription factor Sterol regulatory element binding protein 2 (SREBP2) mRNA activates cholesterol biosynthesis gene to produce HMGCR mRNA. HMGCR increases cholesterol synthesis and CYP7A1 catabolizes cholesterol into bile acids. Betaine supplementation in mammals downregulates SREBP1, SREBP-1c, DGAT1, DGAT2, FAS, and HMGCR, which are required to synthesize and accumulate triglyceride, free fatty acids, and total cholesterol in the liver (106). Thus, betaine improves lipid profile. But this effect is not always consistent, and the effects on lipid profile vary with dietary formulations, dose of betaine, health, and stress (33). In a study, CORT downregulated LXR and CYP27A1 that contributed to cholesterol accumulation in juvenile chickens, but opposite results were demonstrated when CORT-induced embryos were supplemented with betaine (2.5 mg/egg at ED0) earlier (33). During HS, CORT secretion is increased and negatively changes the hepatic lipid profile. Betaine may be effective in alleviating HS, as this study also mimicked the HS by injecting CORT. Without CORT, IOI of betaine alone showed no effect on hepatic cholesterol profile in chickens in that study. The CORT-induced chickens showed CpG hypermethylation. The authors suggested checking the possibility of betaine deposition in eggs through dietary supplementation in laying hens and the efficacy in preventing CORT- or stress-induced cholesterol accumulation in the liver. Later in another study, when laying hens were fed a 0.5% betaine-containing diet for 28 days before egg collection, betaine was transferred to eggs (107). The authors failed to measure the maternal betaine in eggs and offspring liver but measured the epigenetic changes. Maternal betaine decreased the cholesterol biosynthesis enzymes SREBP2 and HMGCR but increased CYP7A1 which breaks down cholesterol to bile acids. Simultaneously, betaine increased hepatic mRNA and protein expression of low-density lipoprotein (LDLP) and reduced mRNA abundance of cholesterol acyltransferase 1 (ACAT1) that mediates cholesterol esterification. Betaine increased CpG methylation on the promoter regions for SREBP2 and ACAT1 but decreased for CYP7A1. Because of CpG methylation, SREBP2 and ACAT1 were downregulated, but CYP7A1 was upregulated. These epigenetic regulations of cholesterol metabolic genes in the offspring initiated by maternal betaine decreased hepatic cholesterol deposition. As the maternal betaine and IOI of betaine in CORT-induced embryo both showed improved regulation of hepatic cholesterol profile, betaine may be a good candidate for commercial IOF practice.

### Carbohydrates

During late embryogenesis, the embryos generate glucose through glycolysis from their glycogen reserve in the liver, through gluconeogenesis from fat, or from protein; initially from amnion albumen, and then from muscle (4).

The IOF of carbohydrates and HMB through amnion on ED 17.5 in broiler chicken eggs increased the liver glycogen by two- to five-fold, elevated breast muscle size from hatch to post-hatch Day 25, enhanced the hatching process, and early development (4). In that study, the increase of the relative mass of the pectoral muscle was ascribed to the probable enhancement of the proliferation of embryonic and neonatal myoblasts and satellite cells due to the IOF. Similarly, IOF with dextrin and HMB-Ca salt in a saline solution increased glycogen reserves, BW, and pectoral muscle weight in both the pre-hatch and post-hatch period; and enhanced myoblast proliferation on ED 19, which remained higher until Day 5 of chicks (32).

The IOF of carbohydrates could play a role in gastrointestinal tract development by triggering goblet cells to produce mucin or increasing the mucin gene expression (35, 108, 109) and by increasing the villus surface area (109).

The IOF of glucose in broiler eggs increased the expression of humoral immune-related genes (IL-6 and IL-10), chicken growth hormone, and IGF-II during late-term embryonic and early post-hatch days (35). In the same experiment, fructose and ribose increased expression of IGF-II, mucin gene, and cellular immune-related genes (IL-2, IFN-γ, IL-12). In turkey, the humoral immune-response was significantly increased after IOF of 1 ml 10% glucose on ED 21 and on ED 25, but the cell-mediated immune response was not altered (36).

The IOF of carbohydrates showed inconsistent results regarding enhancing hatchability. IOF of carbohydrates increased hatchability in a study by Uni et al. (4), decreased hatchability in turkey (36), and delayed the hatching process in broiler chickens (37). The injection of 200 μl of a 0.75% saline solution containing a lower level of carbohydrates (1.5% maltose and 1.5% sucrose, or 2.5% maltose and 2.5% sucrose) increased hatchability, but a high level of carbohydrates (4.5% maltose and 4.5% sucrose) reduced hatchability in domestic pigeons (38). The decreased hatchability could have resulted from hyperglycemia caused by a high amount of carbohydrates injected into the embryo. A dose closer to 1 g/kg glucose can create a hyperglycemic condition in the embryos, leading to heart defects and/or limb defects, disrupted cell proliferation, apoptosis, and finally, may lead to embryonic death and reduced hatchability (39). These can be attributed to the differences in the type and volume of carbohydrates injected. Among other effects, IOF of carbohydrates may lower embryonic metabolism, lower the internal temperature of egg, and increase BW of hatchlings (37).

### Prebiotics

Inulin, fructooligosaccharides (FOS), galactooligosaccharides (GOS), mannanoligosaccharides (MOS), xylooligosaccharides (XOS), and isomaltooligosaccharides are the common prebiotics that are supplemented and found to be beneficial for poultry growth and development (110). Carbohydrate-rich fibers (containing oligosaccharides, polysaccharides, etc.) are fermented in the ceca by microbes and produce short-chain fatty acids (SCFA) along with other metabolites and change the cecal microbial ecology, and promote gut health of host animals (110, 111). However, the fermentation characteristics and changes in the microbial ecology and other gut health markers may vary with carbohydrates or the fiber type (5, 40). Dietary prebiotics act as substrates for particular bacteria, facilitating the bacteria's growth and abundance (40). *In ovo* stimulation by prebiotics causes early-life microbial modulation, which can persistently affect intestinal histomorphology, nutrient uptake, and immunity (41).

Galactooligosaccharide showed a bifidogenic effect by increasing the relative abundance of *Bifidobacterium spp* in the cecum and decreased the number of *Lactobacillus spp* in the ileum by competitive exclusion in 42 days adult broiler chickens (41). In addition, the *in ovo* stimulation increased mRNA expression of cytokine genes (IL-1β, IL-10, IL-12p40) in jejunum and cecum, expression of MUC6 responsible for mucin production in goblet cells in jejunum and cecum, genes of intestinal integrity (CLDN1 and TJAP1), nutrient sensing genes (free fatty acid receptors–FFAR 2 and FFAR4) and showed a variation in the expression of glucose transporter genes (GLUT1, GLUT2, GLUT5) in the large intestine.

The GOS injected *in ovo* can dampen immune responses induced by heat stress in slow-growing chickens. GOS (3.5 mg/egg) delivered at 12 ED downregulated the cytokine (IL-10 and IL-1p40) expression in the chicken under chronic HS and reduced the level of expression in chickens under acute HS, dampened heat-induced Th2 immune responses, and activated oxidative stress responses (CAT and SOD) in chronic HS condition (42). In another study on heat-stressed chickens, IOF of GOS (3.5 mg/egg at ED12) increased BW and daily feed intake, improved FCR, and reduced foot-pad dermatitis. All of these are directly related to HS (43).

Zhang et al. (40) compared the effects of IOF on ED 12.5 of chitooligosaccharide (COS) and chlorella polysaccharide (CPS). They showed that an IOF of 5 mg COS altered the cecal microbiome by increasing the abundance of polysaccharide-utilizing bacteria (*Lactobacillus crispatus, L. johnsonii, L. salivarius, Bacteroides coprophilus, B. coprocola*, and *B. salanitronis*) and by decreasing the opportunistic pathogenic bacteria (*Campylobacter jejuni, Clostridium perfringens, Collinsella stercoris, Corynebacterium efficiens, Fusobacterium mortiferum, Klebsiella unclassified, Shigella boydii*, and *Shigella sonnei*); enriched the pathways of gluconeogenesis, L-isoleucine degradation, L-histidine biosynthesis, and fatty acid biosynthesis; and produced a higher amount of propionic acid. They concluded that COS outperformed CPS and control groups, and the response of the prebiotic COS improved with an increase in age.

*In ovo* techniques are being tried to deliver compounds as an alternative to AGP in poultry diets. A single IOF of Raffinose family oligosaccharides (RFO) in broilers functioned similarly to AGP in the poultry diets (44). In this study, RFO showed a dose-dependent effect in increasing the number of *Bifidobacterium bifidum* and *Lactobacillus acidophilus* (44). In other studies, RFO extracted from the seeds of *Lupinus luteus* L when injected *in ovo* (1.9 mg/egg) did not affect the body weight and FCR but increased meat oxidation and reduced the opportunistic pathogens (*Clostridia)* and coccidia (46), and reduced the hatchability (44). On the contrary, IOI of RFO purified from the seeds of *Lupinus luteus* L increased the BW and FCR in Cobb, Ross, and Hubbard broilers (44). The authors did not identify the reason for the reduced hatchability but pointed out some plausible reasons, such as injection route, type, and dose of bioactive compounds. Later, Berrocoso et al. used RFO with different dose rates (0, 1.5, 3.0, and 4.5 mg RFO/egg) and found that an increased dose of RFO increased the villus height and villus height: crypt depth ratio (45). They also found that the expression levels of CD3 and chB6 genes, which are T cell and B cell marker genes, respectively, were significantly enhanced by IOF of high dose RFO (4.5 mg/egg).

### Probiotics

Probiotics help establish a beneficial gut microbiota that is congenial to the development of gut-associated lymphoid tissues and intestinal integrity (112–115). Probiotics of 17 species are most commonly supplemented in poultry feed as potential AGP (112). The complexity and relative abundance of the microbiome depend on the type of bacteria which colonize and perturb a previously almost empty niche at an early age (116). To harness the benefits of probiotics in prenatal chicks, *in ovo* method for delivering *Lactobacillus reuteri* to amniotic fluid of an embryonic chick was invented and patented for the first time in 1995 (117).

The *B. bifidum* ATTC 29521 and *B. longum* ATTC 15707, 200 μl when using the injected in-yolk route, improved live body weight, BWG, FCR, various hematological indices, and villi height without hampering carcass traits, and liver and renal functions (47). The probiotics produce vitamin B complex, different acids which lowered the pH that increased iron absorption from the small intestine, and the availability of vitamin B and iron escalate erythropoiesis (118).

Avian pathogenic *E. coli* may contaminate the egg through vertical transmission from the laying hen or through a horizontal way (through penetration of the eggshell by the microbe, or because of failure to maintain aseptic *in ovo* delivery practice) (48). A combination of lactic acid bacteria (LAB) of 10 different strains (1 *Lactobacillus johnsonii*, 3 *Weissella confusa*, 2 *Lactobacillus salivarius*, and 1 *Pediococcus parvulus*) was delivered *in ovo* in *E. coli* challenged embryos (48). The LAB significantly increased SCFA-producing Ruminococcaceae bacteria but reduced gram-negative bacteria of the Enterobacteriaceae family (such as *E. coli*) at hatch day and post-hatch Day 7. The IOI of individual probiotic species (*Lactobacillus animalis* ATCC 35046, *Lactobacillus reuteri* ATCC 2837, and *Lactobacillus rhamnosus* ATCC 23272), when injected on ED 18, showed no significant effect on the *E. coli* incidence at post-hatch period (119).

A probiotic culture, FloraMax-B11 (Pacific Vet Group USA^®^ Inc., Fayetteville, AR), consisting of 2 strains of LAB: *Lactobacillus salivarius* and *Pediococcus parvulus* (120) when delivered *in ovo* at ED 18 with HVT vaccine against MD, did not reduce the efficacy of the vaccination and hatchability (49). Furthermore, in a study by Teague et al. (49), IOF of probiotic culture significantly reduced lactose positive Gram-negative bacteria and increased LAB in the gut, increased BW, improved villi surface area in the ileum, and reduced *Salmonella enterica* (SE) incidence during the first 7 days of life in the chickens challenged with SE at the day of hatch.

The pioneer colonizers of the gut at prenatal chickens can alter the protein expression, which regulates the immune and cytoskeleton development (50), and affect the development and diversity of microbiota in the intestine (51). When embryos were injected with different bacterial species (~10e2 cells of *Citrobacter freundii* 97A11 or *Citrobacter spp*. 97A4 or a mixed inoculum of *L. salivarius* and *Pediococcus spp*.) to observe the changes of proteomic expression at DOH, the bacteria-treated groups affected the expression of proteins differently and modulated different canonical pathways (e.g., adherens junction signaling and remodeling, actin cytoskeletal signaling, integrin-linked kinase signaling, calcium signaling, and tight junction signaling) by altering the expression of the proteins (actins, myosins, tubulins, and microtubules) related to those pathways (50). Only the LAB-treated group (*L. salivarius* and *Pediococcus spp*.) enhanced the gluconeogenesis, which is vital to support energy to the embryo and increased antioxidant capacity, whereas the *Citrobacter*-treated groups increased pro-inflammatory reactions and oxidative stress responses. Further, the LAB-treated group showed an upregulation of both peroxiredoxin-1 (PRDX1) and superoxide dismutase-1 (SOD1) which protects cells from free-radical damage caused by ROS and hydrogen peroxide, and ameliorate intestinal oxidative stress by converting hydrogen peroxide into water, respectively (100). Another experiment compared the LAB-treated group to two other groups with similar doses of *in ovo* bacterial inoculation (51). The exposure of LAB to the embryo improved the molecular profiles related to systemic immune processes better than the exposure of two other *Enterobacteriaceae* to the embryo. In addition, *in ovo* LAB exposure to the embryo reduced the *Enterococcaceae* and enhanced the abundance of segmented filamentous bacteria (SFB), a bacterial species named *Candidatus savagella* under the *Clostridiaceae* family, in the mucosa of the lower ileum, which induces maturation of immune system components with aging (51).

*Bacillus spp*.-based probiotics (two *Bacillus amyloliquefaciens* and one *Bacillus subtilis*; 3 × 10e11 spores/g) administered at 18 ED followed by a virulent *E. coli* seeder challenge at ED 19 significantly reduced gram-negative bacteria and thus reduced the risks associated with virulent *E. coli* horizontal transmission, infection of chickens during the hatch, and mortality (52). The probiotic injection increased the bacteria of Lachnospiraceae and Ruminococcaceae family but reduced Enterobacteriaceae. Likewise, *E. faecium*-treated chickens, either delivered through *in ovo* (amnion) or supplemented in feed during post-hatch, increased the protection against Salmonella colonization through competitive exclusion (53).

To evaluate the benefits of IOI of a combination or individual *Enterococcus faecium* and *Lactobacillus animalis* in an industry setting, a total of 2080 eggs divided into four different groups were injected with Invoject^®^ technology (55). This probiotics mixture did not affect hatchability even at high concentrations (10e7 cfu/50 μl *L. animalis* and 10e6 cfu/50 μl *E. faecium*). In a comparative study of probiotics, *E. faecium* showed higher hatchability than *B. subtilis* (96.11 vs. 81.67%) (53).

IOI of *Bacillus subtilis* ATCC 6051 (10e3 to 10e6 cfu/50 μl/egg) significantly reduced the hatch of fertile eggs and increased mortality of embryos (56). In the same experiment, *L. acidophilus* and *B. animalis* showed no effect on hatchability. The authors opined that the nutrient competition between the *Bacillus subtilis* for sporulation and embryo for the hatch and the production of bacteriocins and enzymes (protease, amylase, and cellulose) might lead to the reduced hatch rate. A study showed that the effect of IOI of *B. subtilits* on the hatchability is serotype dependent (121). In that study, *B. subtilis* ATCC 8473 and *B. subtilis* ATCC 9466 did not affect the hatchability, but *B. subtilis* ATCC 6051 reduced hatchability to 17.3%.

The probiotic strains could be selected for IOF based on flux balance analysis which will help to generate the metabolites at the optimal level. As a very small number of microbes can be injected, the amount should be of more efficacious strains to change the metabolic atmosphere of the embryo gut.

### Synbiotics

The IOI of bioactive compounds can induce epigenetic change by modulating embryonic gut microbiota. IOI of *L. plantarum* and RFO as synbiotics improved metabolic gene expression (downregulated ANGPTL4, upregulated NR4A3) in the liver, and the change was better than IOI of other synbiotics (*L. salivarius* and GOS) (57). But the Lactobacillus synbiotics in both combinations showed no change in the expression of immune-related genes (SYK and KLHL6) in the liver. But when a multi-strain Lactobacilli mixture (*Lactobacillus salivarius, L. reuteri, L. crispatus*, and *L. johnsonii*) was administered *in ovo*, the injection caused an elevated expression of cytokines in the spleen but a downregulated expression in the cecal tonsils (54). When birds were immunized with KLH (keyhole limpet hemocyanin), the Lactobacilli-treated groups showed enhanced serum IgG and IgM responses against KLH, which was an indication of the inability of the Lactobacilli to stimulate the T cells in the spleen in non-immunized chickens. However, sheep red blood cell (SRBC) immunization did not affect antibody production.

Duan et al. (122) studied the effect of IOF of *L. plantarum* and astragalus polysaccharide at ED 18.5. The IOF of this synbiotic improved the growth performance, immunity, and morphology of the small intestine and cecal microflora.

Inulin works better when used as a synbiotic (a combination with *L. lactis* subsp. *Lactis* IBB2955, and inulin) than as a prebiotic alone (123). This synbiotic was found to be conducive to improve the expression of the genes related to energy metabolism and immune responses in the spleen and cecal tonsils. The study by Dunislawska et al. (123) showed that these transcriptomic responses to *in ovo* stimulation last until post-hatch Day 35.

### Hormones

Different hormones have been used *in ovo* with variable success. With an intent to find the effect of IOD of CRH in broiler embryos, 0.1, 1, or 2 μg of CRH was injected either through the air cell, albumen, or amniotic fluid on ED 18 in Cobb eggs or through the albumen daily from ED 10 to ED 18 (58). When repeated, the experiments did not consistently affect hatching time and were considered an unfeasible method in improving hatchery productivity.

An ontogenetic study (124) of thyrotropin-releasing hormone (TRH) concentrations in the brain of chicken embryos showed that TRH concentration increased toward the end of incubation, and the rise in hypothalamic TRH was almost 15 times from ED 14 to Day 1 of chicks. But extrahypothalamic and hypophyseal TRH levels decreased toward hatching; pituitary TRH started declining from its highest concentration of ED 14-ED 16 to five- to ten-fold lower at the end of incubation. The authors opined the tissue-specific fluctuations to the late maturation of the hypothalamic control and suggested that the IOD of TRH may compensate for that situation. TRH regulates the level of triiodothyronine (T3) and thyroxine (T4) production, and vice versa. The IOI of TRH improved hatchability and elevated embryonic blood plasma T3 and T4 in turkey (59) when injected either through the air cell membrane or through the small end of the eggs. Other than the variation due to the injection route, the study showed that the embryos of different strains of turkeys might respond differently to TRH.

IOI of 0.2 or 1 μg of corticosterone on ED 11 suppressed the post-hatch growth rate during the first 21 days of life, increased the aggressive behaviors (Day 28), elevated plasma corticosterone (Day 42), and downregulated hypothalamic expression of arginine vasotocin and CRH (60).

In a study, 65 ng of thyroxine was injected at ED 18, and the eggshell temperature was manipulated by exposing broiler chicken eggs to 15°C for 1 h on ED 11, 13, 15, and 17 (61). The injection of thyroxine and manipulation of temperature increased the number of first-grade chicks, decreased yolk sac weight, but increased body weight at hatch. Moreover, these chickens showed a reduced mortality rate when exposed to ascites-inducing conditions during 22–42 days post-hatch and developed ascites.

### CpG Oligodeoxynucleotides

A chick's immune system is weak and incapable of defending against bacterial and viral infections at its first 2 weeks of age, which makes chicks prone to diseases (125). The outcomes from the pathogenic infection can be prevented by administering immuno-modulating agents such as synthetic oligodeoxynucleotides with non-methylated cytosine-guanine motifs (CpG ODN), poly[di(sodium carboxylatoethylphenox-y)phosphazene] (PCEP), PCPP, and loxoribine. CpG ODN binds TLR 21 of macrophages and dendritic cells and stimulates these cells to secrete cytokines and other effectors such as nitric oxide, IFN-α, IFN-γ, IL-1β, IL-6, and TNF-α (62, 64, 65, 126). This immunomodulator can activate the innate immunity rapidly, and its effect prevails for a long time (65, 127).

As a first attempt to examine the immuno-protective effect against intracellular *S. typhimurium*, IOI of 50 μg CpG-ODN at 18 ED was followed by the ST challenge (66). The injection increased the survival rate and decreased bacterial loads and pathology in the challenged birds. Similarly, the IOI of this immuno-stimulant showed immuno-protection against a lethal challenge of *E. coli* in neonatal chicks (63, 126) and SE (65). Allan et al. (62) compared the antibacterial protection against *E. coli* in neonatal chicks of CpG-ODN with polyinosinic:polycytidylic acid, cyclic polyphosphazene, and loxoribine. Their CpG injection resulted in a more than 80% survival rate from yolk sac infection and reduced embryo chick mortality.

In a study, 100 μl PBS solution containing 25 μg CpG-ODN was injected per embryo, the given CpG induced immune response against bacterial infection (65). At 2 days post-hatch, blood collected from treated birds had shown increased degranulation in heat-killed SE- or live SE-stimulated heterophils. But there was no effect in heterophil oxidative burst in either heat-killed SE- or phorbol myristate acetate-stimulated heterophils. In the same study, later, some chickens were challenged with *S. enterica* serovar *Enteritidis* on post-hatch Day 10, and cecal contents were collected on Day 16 to test the lasting effect of CpG. Owing to the increased heterophil function as an indicator of the lasting effect of CpG, there was an astonishing declination of SE by >10-fold in cecal contents.

Gunawardana et al. compared the efficacy of CpG-ODN formulated with carbon nanotubes or liposomes against unformulated CpG-ODNs to induce protection against *E. coli* infection (63). Embryonated eggs received 50 μg of either CNT-CpG-ODN, LSC-CpG-ODN, or unformulated CpG-ODN at ED18 (63). Formulated CpG-ODNs increased the survival rate, decreased bacterial loads, and clinical scores at *E. coli* challenged neonatal chickens. Other CpG formulated with polyphosphazenes such as Poly [di (carboxy-latophenoxy phosphazene) or PCPP, and Poly [di (sodium carboxylatoethylphenoxy) phosphazene] or PCEP can enhance CpG-induced immunity to create protection against bacterial infection in neonatal chickens (67). CpG may develop this protection against *E. coli* by enriching immunological niches in the spleen and lungs (69). This supplementation provided a higher and prolonged expression of lipopolysaccharide-induced tumor necrosis factor (LITAF), a pro-inflammatory cytokine, which improved the immune functions of the spleen and lungs in a similar study. As CpG activates TLR21 and increases LITAF, the author suggested TLR21-LITAF-mediated immune enrichment may increase the therapeutic application of CpG.

The antiviral role of CpG in chicken embryos was first demonstrated in a study where IOI of CpG ODN 2007 (B-class) at ED 18 showed increased expression of IFN-γ, interleukin (IL)-1β, IL-6, IL-8, 2′-5′-oligoadenyl synthetase A (OASA) mRNA in embryonic spleen tissue; and lessened the infectious bronchitis virus (IBV) replication in IBV challenged embryos (64). This supplementation showed antiviral activity by suppressing IBV N gene mRNA expression in various embryonic tissues. In addition, some interferon stimulating genes activate IFN-γ, IFN-γ expresses OASA proteins. Subsequently, OASA activation triggers RNAase L that inactivates viral mRNA and host cell 28S rRNA and prevents viral replication (64).

Encapsulated CpG was used as an adjuvant to check the effects of this in enhancing the efficacy of the herpes virus of turkey (HVT) vaccine for boosting immunity against Marek's disease (MD) (68). ECpG moderately improved the efficiency of HVT and reduced tumor incidence and MD virus load.

### Other *in ovo* Supplements

Most commonly used supplements delivered through *in ovo* are discussed in this review. The review by Peebles (89) has discussed some other *in ovo* supplements.

Ncho et al. (128) reported that IOI of γ-amino-butyric acid (GABA), a non-protein amino acid, can increase total antioxidant capacity and reduce the adverse effects of heat stress by downregulating HSP70 gene expression.

Micromineral combination shows beneficial effects on embryo and post-hatch chicks. Oliveria et al. (129) showed that IOF of a combination of zinc, manganese, and copper enhances bone mineralization. However, the research articles on the use of nanoparticle-based *in ovo* delivery is scarce. As the interest in nanotechnology is increasing, the research related to IOF of nanoparticle-based minerals can contribute to the poultry industry. Such as, calcium carbonate nanoparticles at a dose rate of 500μg/mL can improve bone mineralization during embryo development (130).

Saleh et al. (131) aimed to investigate the effect of IOI of Clenbuterol on the embryos and post-hatch chickens. Clenbuterol *in ovo* reduced abdominal fat deposition. Moreover, this increased the growth efficiency by downregulating the myostatin gene and increasing protein synthesis.

## Challenges and Future Potential

The continuous development and improvement of *in ovo* technology have established a new scope for perinatal nutrition, allowing and creating new challenges and opportunities for poultry researchers to optimize poultry production. The administration of digestible nutrients *in ovo* can improve bird quality, increased glycogen reserves, fast development of the total digestive tract, superior skeletal health, better muscle growth rate, higher body weight gain, improved feed conversion, and enhanced immune function (89). However, the main limitations still are associated with embryo development and nutrient metabolism. Another question is a limitation in the preparation of supplements that fits the specific needs of the individual bird. Future early nutrition would be feeding complex nutrients and supplements that would replace feed additives and supplements in the post-hatch feed and would be more beneficial to the poultry industry. Also, there is a lack of standardized protocols for *in ovo* delivery of compounds into the eggs, which affects the response of IOF program. For example, a carrier or vehicle used as a diluent for the biological substances in IOD can reduce hatchability up to 10% compared with non-injected control (53). Also, the route, dose, method, and injection time severely affect the embryonic growth and post-hatch performance of chickens. Thus, there is a need to establish appropriate protocols for diluent(s), time, and route for the IOD of compounds.

Currently, researchers are interested in injecting one nutrient through *in ovo* techniques. But providing one specific nutrient to prevent one pathogenic infection is not economical and may leave the chickens susceptible to other microbial infections (132). But, adding nutrients to hatching eggs helps the chicken embryo get the required nutrients for embryonic and neonatal development, which can be considered a congruent biological nutritional support to the fetus in the mammals (4). Also, the response to supplements may vary with the strains of the chickens. Therefore, the investigations should focus on finding the optimal doses of supplements, their effects on immunomodulation, gut microbiota, etc.

Despite the challenges yet to be overcome, the *in ovo* technique has shown great potential for commercial adaption in the poultry industry. The automation of *in ovo* technology has reduced the labor cost, the risks of pathogenic contamination, and wastage of vaccines and bioactive inoculants as the *in ovo* delivery machines can self-sterilize, detect and inoculate only the viable embryos (133).

## Conclusion

*In ovo* feeding is one of the latest and successful methods to feed embryos for improved performance and health during pre-hatch and post-hatch periods. It is crucial as it provides the chicks with sufficient nutrients and supplements during the lag period (from hatch to first feed in the production farm). Currently used materials for *in ovo* feeding include nutrients like glucose, amino acids, and vitamins, and supplements like probiotics, prebiotics, exogenous enzymes, hormones, vaccines, drugs, and other nutraceuticals. Several studies have shown that the *in ovo* injection of different compounds can increase the number of quality hatched chicks, decrease yolk sac weight, increase body weight at hatch and post-hatch period, reduce mortality, increase carcass yield, improve metabolism of nutrients, improve gut morphology, change blood histology, modify the regulation of transcription of different genes, boost immunity, and protect against harmful gut microbes through competitive exclusion. *In ovo* feeding of pre-, pro-, and synbiotics and CpG-ODN can reduce bacterial loads in the gut and reduce pathological conditions caused by bacteria in the challenged birds. Some studies have been done to evaluate the effect of a combination of compounds where the conclusion cannot be drawn which compound could be credited for the beneficial effects. Though the combination of supplements achieves a beneficial effect, it is important to understand the specific contribution of each ingredient in achieving the combined effect. This technique has been found to be promising in increasing the body weight of chicks on the day of hatch and at certain ages during the bird's life. Some bioactive compounds, when supplemented *in ovo*, showed beneficial effects akin to AGP. These compounds can be opted for *in ovo* inoculation to initiate the effect earlier in life. But to replace AGP throughout the post-hatch period, the effects of *in ovo* injection should be ensured through long-time trials expanding from the perinatal period to the marketable age of the poultry. The outcomes of an *in ovo* injection of a biological compound may vary according to species, strain, time, and route of injection. For commercial adaptation, the optimum injection procedures must be established to get reproducible results and broader application in commercial production systems.

## Author Contributions

RD reviewed the literature and wrote the draft of the manuscript. PM reviewed and contributed to writing the manuscript. RJ conceptualized the paper, decided the review topic, contributed to writing, reviewed, and finalized the manuscript. All authors contributed to the article and approved the submitted version.

## Conflict of Interest

The authors declare that the research was conducted in the absence of any commercial or financial relationships that could be construed as a potential conflict of interest.

## Publisher's Note

All claims expressed in this article are solely those of the authors and do not necessarily represent those of their affiliated organizations, or those of the publisher, the editors and the reviewers. Any product that may be evaluated in this article, or claim that may be made by its manufacturer, is not guaranteed or endorsed by the publisher.
